# Melasolv induces melanosome autophagy to inhibit pigmentation in B16F1 cells

**DOI:** 10.1371/journal.pone.0239019

**Published:** 2020-09-17

**Authors:** Hyun Jun Park, Doo Sin Jo, Hyunjung Choi, Ji-Eun Bae, Na Yeon Park, Joon Bum Kim, Ji Yeon Choi, Yong Hwan Kim, Gyeong Seok Oh, Jeong Ho Chang, Hyoung-June Kim, Dong-Hyung Cho

**Affiliations:** 1 School of Life Sciences, Kyungpook National University, Daegu, Republic of Korea; 2 Brain Science and Engineering Institute, Kyungpook National University, Daegu, South Korea; 3 R&D Unit, AmorePacific Corporation, Yongin, Gyeonggi-do, Republic of Korea; 4 Department of Biology Education, Kyungpook National University, Daegu, South Korea; Sungkyunkwan University, REPUBLIC OF KOREA

## Abstract

The melanosome is a specialized membrane-bound organelle that is involved in melanin synthesis, storage, and transportation. In contrast to melanosome biogenesis, the processes underlying melanosome degradation remain largely unknown. Autophagy is a process that promotes degradation of intracellular components’ cooperative process between autophagosomes and lysosomes, and its role for process of melanosome degradation remains unclear. Here, we assessed the regulation of autophagy and its contributions to depigmentation associated with Melasolv (3,4,5-trimethoxycinnamate thymol ester). B16F1 cells-treated with Melasolv suppressed the α-MSH-stimulated increase of melanin content and resulted in the activation of autophagy. However, introduction of bafilomycin A1 strongly suppressed melanosome degradation in Melasolv-treated cells. Furthermore, inhibition of autophagy by ATG5 resulted in significant suppression of Melasolv-mediated depigmentation in α-MSH-treated cells. Taken together, our results suggest that treatment with Melasolv inhibits skin pigmentation by promoting melanosome degradation via autophagy activation.

## Introduction

Organelles are specialized intracellular structures that perform specific critical roles for cell function and survival; the number of organelles can be modulated in response to specific functional and environmental needs [1]. Autophagy is a pathway that promotes intracellular degradation of large protein aggregates and damaged organelles. During autophagy, targeted cytosolic constituents are isolated within double-membrane vesicles called autophagosomes; these eventually fuse with lysosomes and undergo degradation [2, 3]. The acidic pH in the lumens of these organelles is optimal for the activities of lysosomal hydrolytic enzymes that degrade cellular components [2, 3]. Various autophagy-related genes (ATGs), including ATG5 and ATG7, mediate the actions of autophagic pathways. Recent studies have shown that target organelles can be eliminated via organelle-specific autophagy, for example, mitophagy, which is a unique pathway that promotes mitochondrial autophagy [4, 5].

Melanosomes are unique organelles that are responsible for color and photoprotection in the skin and promote diverse cellular processes including melanogenesis, a complex regulatory process that includes melanin production, transportation, and release [6]. Several of the proteins that regulate melanogenesis have been identified. Microphthalmia-associated transcription factor (MITF) is a master regulator of the expression of melanogenesis-related proteins that include tyrosinase and tyrosinase-related protein 1/2 [7, 8]. As tyrosinase activity is very important in the control of melanin synthesis, various approaches focused on inhibiting these melanogenic enzymes have been used to treat skin hyperpigmentation [9, 10]. The MITF family includes four distinct genes: MITF, the transcription factor EB (TFEB), TFE3, and TFEC [11, 12]. TFEB is a major transcriptional regulator of autophagy, as it promotes the expression of genes required for autophagosome formation including the ATGs [12, 13].

Recently, our group and others have reported that autophagy regulates melanogenesis in both melanocytes and keratinocytes [14–17]. Although autophagy may also contribute to skin color via its role in regulating melanin degradation, the detailed mechanism has not been clearly defined. A full understanding of the mechanisms underlying melanogenesis may help to explain pigmentation dysregulation disorders and likewise to facilitate the development of important cosmetic strategies. In this study, we have developed a monitoring system for melanosome-selective autophagy, (melanophagy), and also evaluated the effects of 3,4,5-trimethoxycinnamate thymol ester (Melasolv) on melanophagy in mouse melanoma B16F1 cells.

## Materials and methods

### Reagents and plasmid

Melasolv was synthesized by Amorepacific Research Group, as described previously [18]. ARP101, Torin1 were purchased from TOCRIS (Bristol, UK). Biochemicals α-melanocyte-stimulating hormone (α-MSH) and bafilomycin A1 were purchased from Sigma-Aldrich (St. Louis, MO, USA). The expression plasmid pEGFP-LC3 was a gift from Tamotsu Yoshimori (Osaka University, Japan) [19]. Plasmids pEGFP-TFEB, pmRFP-EGFP-LC3 (21074), and pEGFP-two-pore channel (TPC2) (80153) were purchased from Addgene (Watertown, MA, USA). For pcDNA/TPC2-mRFP-EGFP plasmid construction, PCR-amplified products TPC2 and mRFP-EGFP were individually subcloned into pcDNA3.1/Myc-His(−)A. A validated siRNA for mouse Atg5 siRNA(5‘-ACCGGAAACUCAUGGAAUA-3‘) and scrambled control siRNA (5‘-CCUACGCCACCAAUUUCGU-3‘) were synthesized by Bioneer (Daejeon, Korea).

### Cell culture

Cells from the B16F1 melanoma line, which were obtained from ATCC, were cultured at 37°C in a 5% CO2 incubator and maintained in Dulbecco’s Modified Eagle’s Medium containing 10% fetal bovine serum and 1% penicillin/streptomycin (Invitrogen, Carlsbad, CA, USA). To generate stable cell lines, B16F1 melanoma cells were transfected with pEGFP-LC3 (B16F1/GFP-LC3), pcDNA/TPC2-mRFP-EGFP (B16F1/TPC2-mRFP-EGFP), or pEGFP-TFEB (B16F1/TFEB) with Lipofectamine 2000 according to the manufacturer’s protocol (Invitrogen). Stable transfectants were selected by growth in a selection medium containing 1.25 mg/ml of G418 (Invitrogen) for 10 days, and colonies derived from single transfected cells were isolated. Stable clones were selected by visualization under a fluorescence microscope.

### Melanin content assay

Melanin content determination was performed using a slight modification of a previously described method [14]. To measure the melanin contents, B16F1 cells were harvested by trypsinization and dissolved in solubilization buffer at 100°C for 30 min. Relative melanin content was determined by measuring at 405 nm using an ELISA plate reader (PerkinElmer, Victor X3).

### Autophagy analysis and melanophagy assay

B16F1/GFP-LC3 cells were treated with Melasolv (10 μg/ml) or ARP 101 (10 μM). Autophagy was determined by the number of cells that displayed GFP-LC3 punctate structures indicative of autophagosomes via fluorescence microscopy (IX71, Olympus, Japan). B16F1/TPC2-mRFP-EGFP cells were seeded on coverslips in 12-well plates. At 70% confluence, the cells were treated with Melasolv (10 μg/ml) or left untreated in the presence or absence of bafilomycin A1 (5 nM) for 12 h. The cells were then washed with phosphate-buffered saline (PBS, pH 7.4), fixed with 4% paraformaldehyde at room temperature for 20 min, and then washed with PBS. After mounting with coverslips, cells were evaluated under a confocal microscope. The number of cells with red punctate structures was counted; the findings were presented as a percentage of total cells from counts of 200 cells.

### Western blotting

All lysates were prepared with 2× Laemmli sample buffer (Bio-Rad, Hercules, CA, USA). Total protein was measured using the Bradford assay (Bio-Rad) according to the manufacturer’s instruction. Samples were separated by SDS-polyacrylamide gel electrophoresis and transferred to PVDF membrane. After blocking with 4% skim milk in tris-buffered saline supplemented with Tween-20, the membrane was incubated with primary antibodies including anti-MITF (MS-771-P1; Neomarkers), anti-TYR (a gift from Amorepacific Research Group), anti-LC3 (NB100-2220), anti-ATG5 (NB110-53818) and anti-actin (NB600-501; NOVUS Biologicals, Littleton, CO, USA), and anti-GFP (sc-9996; Santa Cruz Biotechnology, Dallas, TX, USA). For protein detection, the membranes were incubated with HRP-conjugated secondary antibodies (Pierce, Rockford, IL USA).

### Confocal microscopy

B16F1/TPC2-mRFP-GFP cells were plated on glass-bottom dishes and treated with α-MSH (1 μM; M4135, Sigma, St. Louis, MO, USA) for 48 h and Melasolv for 24 h. The cells were then washed with PBS and fixed with 4% paraformaldehyde for 20 min. Then, the fluorescence images of TPC2-mRFP-EGFP cells were obtained using a LSM 700 laser-scanning confocal microscope (LSM 700; Objective C-Apochromat 40x/1.2 W Corr UV-VIS-IR M27; Carl Zeiss, Thornwood, NY, USA) and processed using ZEISS Zen Software.

### Statistical analysis

Data were obtained from at least three independent experiments and presented as means ± SEM. Statistical evaluation of the results was performed with one-way ANOVA. Data were considered significant at a value of p<0.05.

## Results

### Melasolv activates autophagy in B16F1 cells

**Fig 1:**
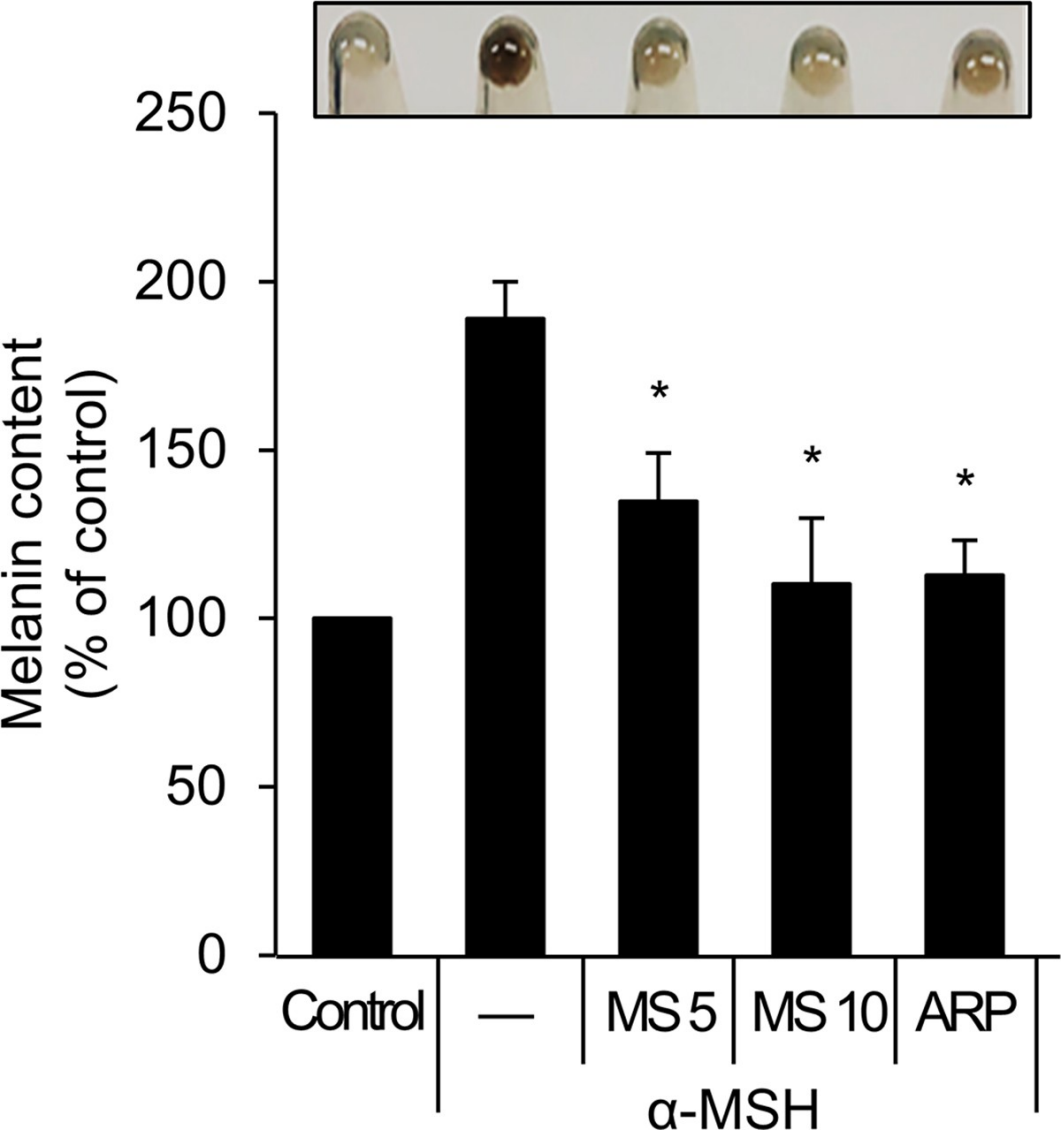
Melasolv induces depigmentation in B16F1 cells. B16F1 cells were pre-treated with α-MSH (1 μM) for 48 h and then further incubated with Melasolv [MS 5, 10 (5 μg/ml, 10 μg/ml)] or ARP101 (ARP, 10 μM) for 24 h. Then the cell pellets were collected to determine melanin content.

**Fig 2:**
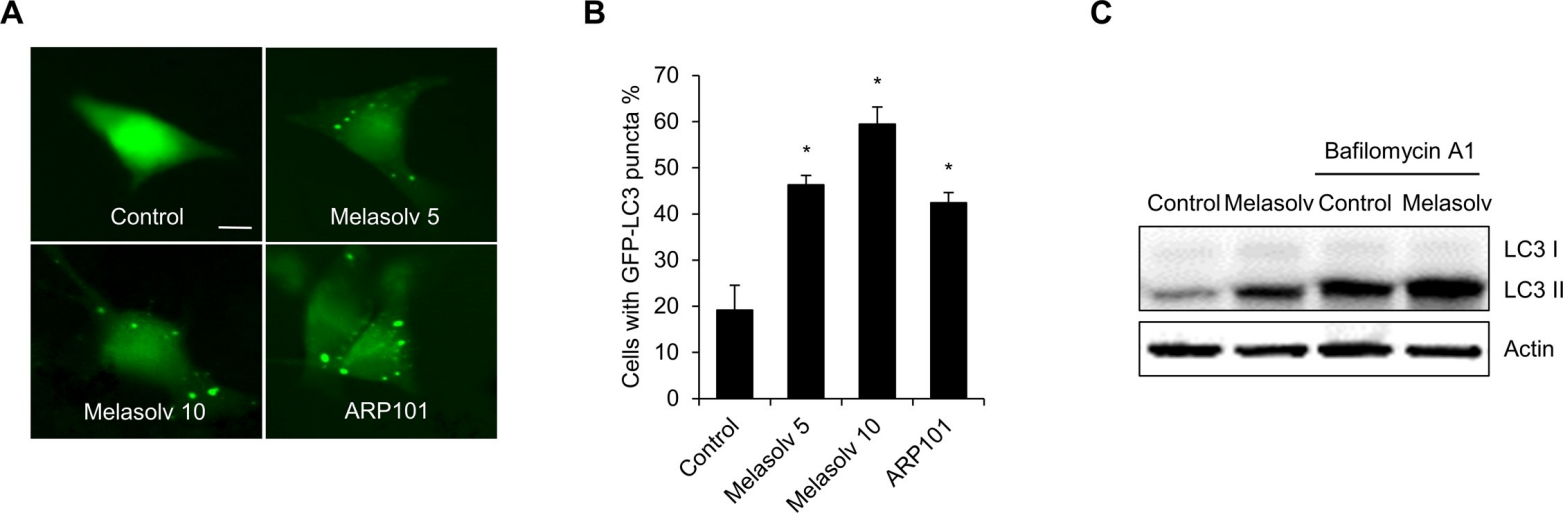
Melasolv induces autophagy activation in B16F1 cells. (A, B) B16F1/GFP-LC3 cells were treated with either Melasolv (5–10 μg/ml) or ARP101 (10 μM). After 24 h treatment, the cells were fixed for fluorescence imaging (A). The scale bar indicates 10 μm. The cells with autophagy activation were determined by counting punctate GFP-LC3 dots under a fluorescence microscope (B). Data were obtained from about 200 cells per group and experiments were repeated at least three times. (* p<0.05, SEM, n = 3). (C) B16F1 cells were treated with Melasolv (10 μg/ml) in the presence or absence of bafilomycin A1 (5 nM) for 24 h. The level of LC3 protein was then assessed by Western blotting.

**Fig 3:**
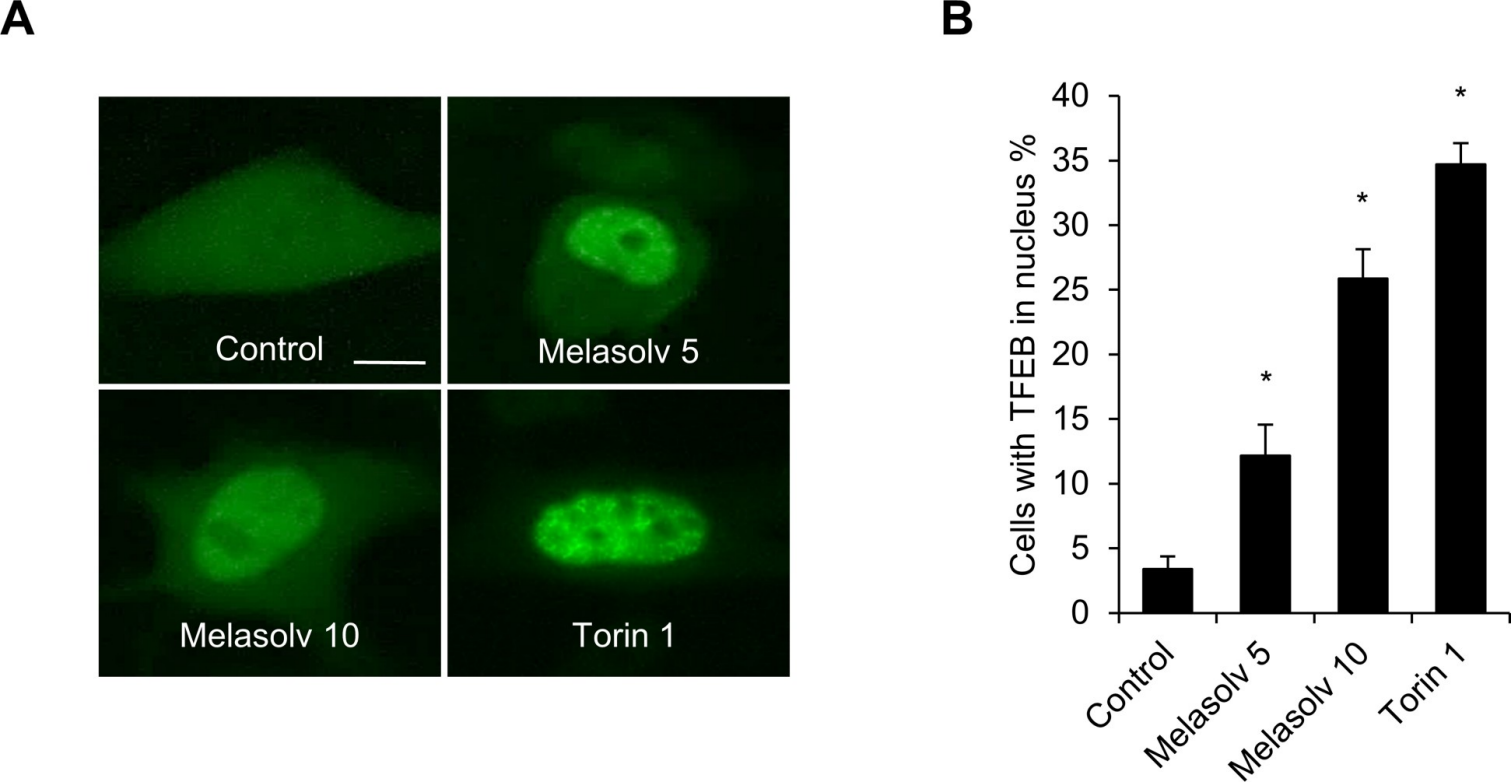
Melasolv induces translocation of TFEB in B16F1 cells. (A, B) B16F1/GFP-TFEB cells were treated with either Melasolv (5–10 μg/ml) or incubated with Torin1 (0.25 μM for 1 h). The cells were fixed and imaged by a fluorescent microscopy (A). And nuclear localization of GFP-TFEB was analyzed (B). Scale bar, 10 μm. Data were obtained from about 200 cells per group and experiments were repeated at least three times. (* p<0.05, SEM, n = 3).

From a previous biochemical screening, we identified Melasolv as the most potent of the depigmenting agents that does not promote cytotoxicity [18]. Melasolv inhibits pigmentation in various experimental models, including α-MSH-treated B16F1 cells, primary normal human melanocytes, and a human skin equivalent system [20]. To confirm the whitening effect characteristic of Melasolv, B16F1 cells stimulated with α-MSH were treated with Melasolv, and the melanin content was analyzed. Consistent with the previous report, Melasolv efficiently suppressed melanogenesis in B16F1 cells, despite the strong melanogenic stimulus provided by α-MSH (Fig 1). As autophagy is one means to inhibit skin pigmentation, we further examined whether Melasolv activates cellular autophagy pathways in these cells. B16F1 cells stably expressing GFP-LC3 (the autophagy activation marker, microtubule-associated protein 1A/1B-light chain 3) were treated with either Melasolv or the matrix metalloproteinase two inhibitor, ARP 101, a potent inducer of autophagy [14]. As shown in Fig 2A and 2B, the formation of intracellular punctate deposits containing GFP-LC3 protein underwent dramatic increase in response to treatment with Melasolv. To examine autophagy flux in response to treatment, B16F1 cells were incubated with Melasolv with or without the autophagosome–lysosome fusion inhibitor, bafilomycin A1. The level of immunoreactive LC3 was higher in cells treated with Melasolv and bafilomycin A1 than in cells treated with either of these compounds alone, suggesting that Melasolv is a potent autophagy inducer (Fig 2C). Earlier studies have reported that TFEB, which was phosphorylated in response to inhibition of mammalian target of rapamycin (mTOR), is retained in the cytoplasm, whereas dephosphorylated TFEB undergoes translocation to the nucleus to induce the transcription of autophagy-associated target genes including various ATGs [12]. Here, we found that treatment with Melasolv induces nuclear translocation of TFEB to an extent similar to that induced by Torin1, a potent mTOR inhibitor in B16F1 cells (Fig 3A and 3B). Taken together, these results indicate that Melasolv activates autophagy in B16F1 cells.

### Melasolv induces depigmentation by increasing melanophagy in B16F1 cells

**Fig 4:**
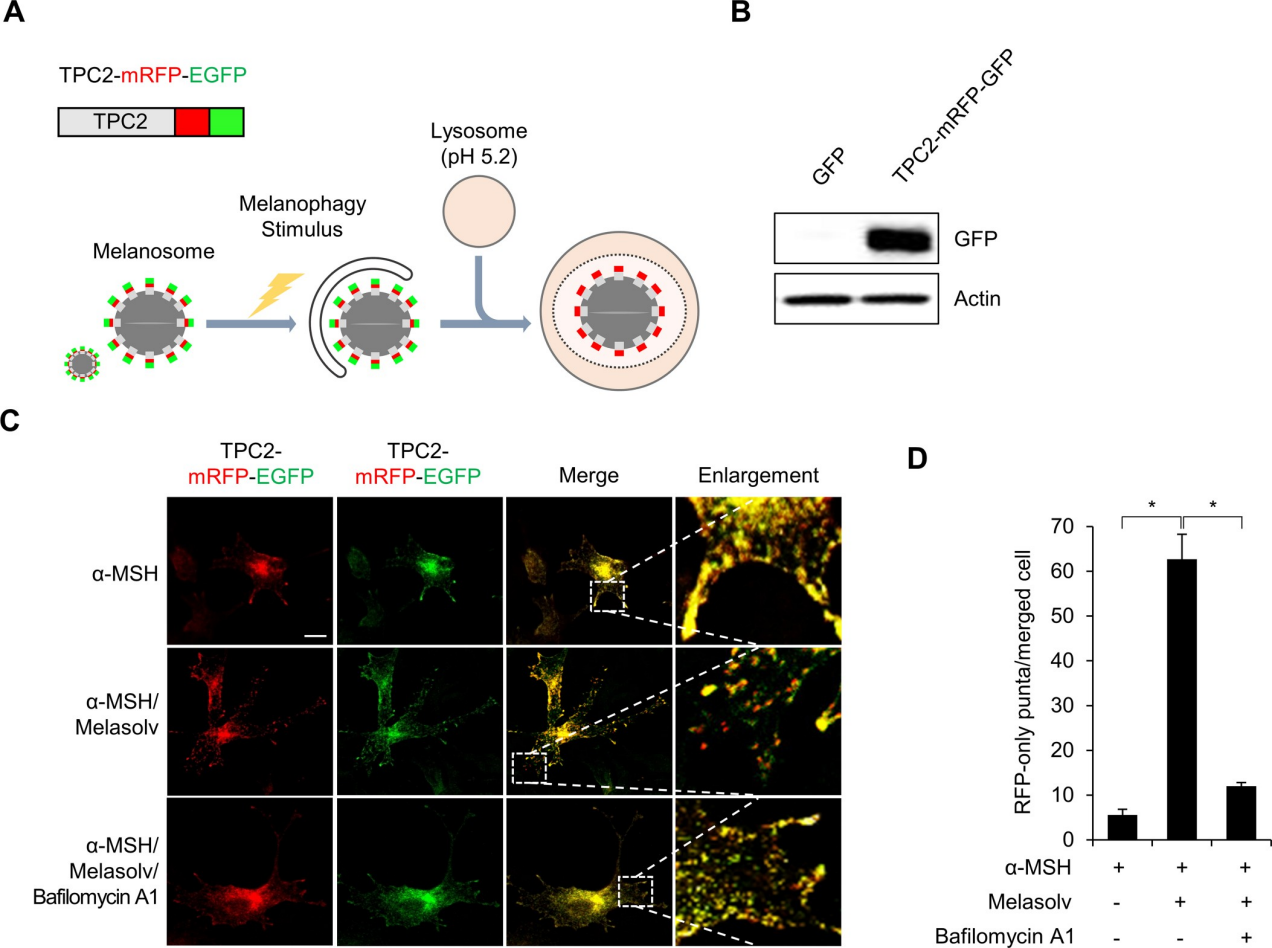
Melasolv induces depigmentation by increasing melanophagy in B16F1 cells. (A, B) Schematic representations of the measurement of autophagic flux of melanosome using TPC2-mRFP-EGFP. Expression of TPC2-mRFP-EGFP in B16F1/TPC2-mRFP-EGFP cells was confirmed by Western blotting with anti-GFP antibody (B). (C, D) B16F1/TPC2-mRFP-EGFP cells were pre-treated with α-MSH (1 μM) for 48 h and then further incubated with melasolv (10 μg/ml) with or without bafilomycin A1 (5 nM) for 24 h. (C) The cells were fixed and distribution of TPC2-mRFP-EGFP was imaged by a confocal microscopy. The scale bar indicates 10 μm. (D) Cells presenting RFP-only puncta were quantified with the merged images (* p<0.05, SEM, n = 3).

To determine whether Melasolv is a potent inducer of melanophagy, we developed a monitoring system featuring TPC2 protein followed by tandem fluorescent tags (TPC2-mRFP-EGFP). The basic principle of this assay is based on different pH stability of the green (GFP) and red (RFP) fluorescent proteins. The acidic environment within the lysosome (pH 5.2) quenches the fluorescent signal of EGFP with only minimal impact on mRFP [21]. During melanophagy, targeted melanosomes are selectively engulfed by autophagosomes that are delivered to the lysosomes for degradation. In the lysosome, the acid-sensitive GFP signal is quenched, whereas the stable RFP signal remains, suggesting a melanophagic process (Fig 4A and 4B). TPC2 is primarily expressed in the melanosome-limiting membranes of melanocytes [22–24]. As such, we generated a stable B16F1 cell line that expresses TPC2-mRFP-EGFP (B16F1/TPC2-mRFP-EGFP). To examine melanophagy with this monitoring system, B16F1/TPC2-mRFP-EGFP cells stimulated with α-MSH were treated with Melasolv. As shown in Fig 4C and 4D, treatment with Melasolv drastically increased the RFP-only positive signals (red dots). Whereas addition of bafilomycin A1 strongly suppressed the generation of RFP-labeled melanosomes (Fig 4D).

**Fig 5:**
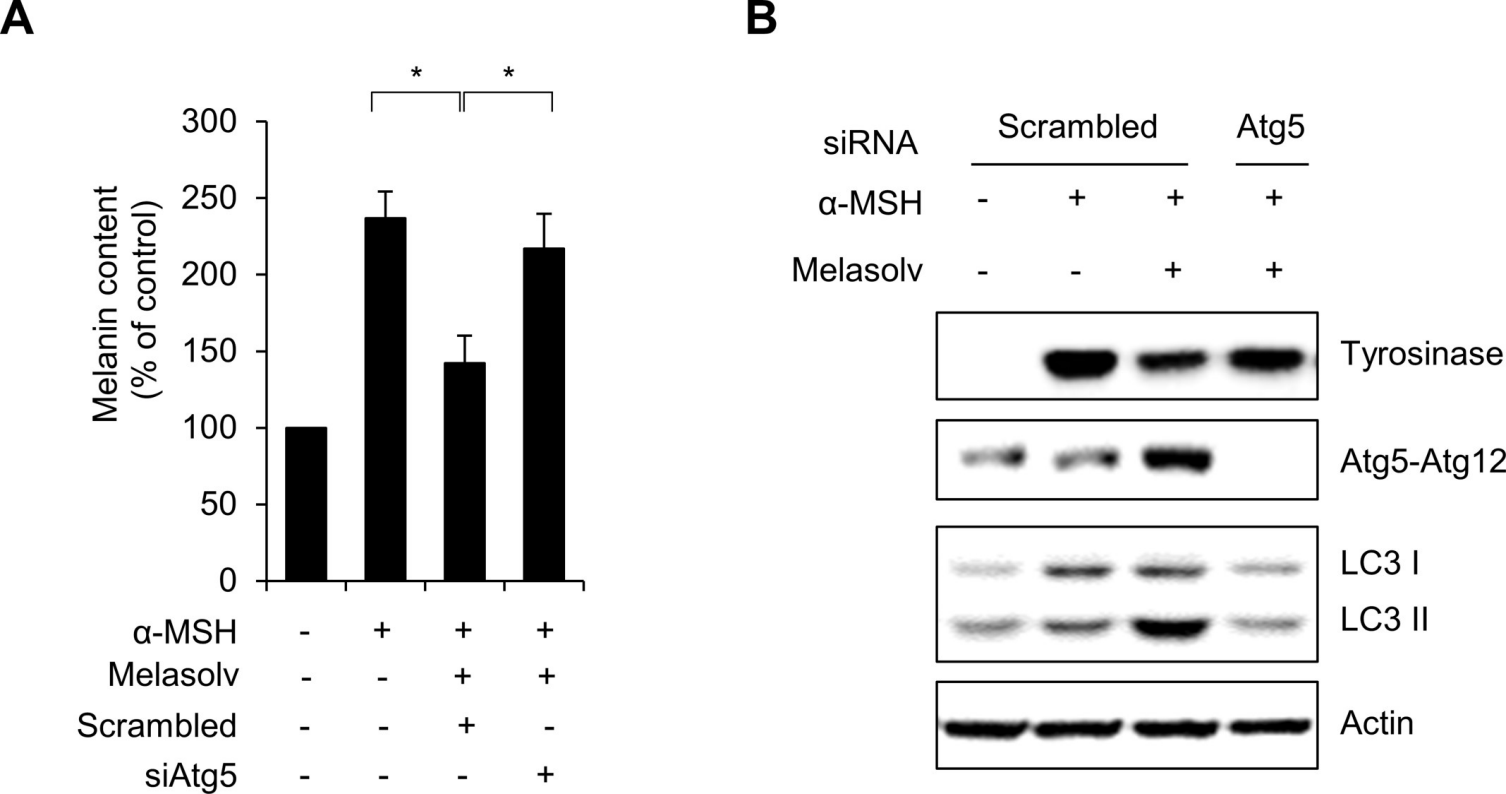
Loss of ATG5 inhibits depigmentation activity of Melasolv in B16F1 cells. (A, B) B16F1 cells were transfected with scrambled control siRNA (Sc) or Atg5 siRNA (siAtg5). After 1 day, the cells were further treated with α-MSH (1 μM) for 48 h, and exposed to melasolv (10 μg/ml) for additional 24 h. (A) After 96 h post-transfection, the cells were harvested and lysed to measure melanin contents. (B) The protein expression was assessed by Western blotting using indicated antibodies. (* p<0.05, SEM, n = 3).

As activation of autophagy led to decrease in melanin content, we further addressed the issue of Melasolv-induced activation of autophagy on pigmentation in B16F1 cells. Consistent with our hypothesis, we found that inhibition of autophagy by targeted suppression of ATG5 suppressed Melasolv-mediated depigmentation in α-MSH-stimulated B16F1 cells (Fig 5A). Furthermore, suppression of ATG5 restored the levels of immunoreactive tyrosinase, which had been suppressed in response to treatment with Melasolv in α-MSH-stimulated cells (Fig 5B). Taken together, these results suggest that Melasolv induces melanosome degradation via activation of autophagy.

## Discussion and conclusion

Previous screening of our synthetic compound library resulted in the identification of Melasolv as a potent depigmenting agent; we also described the anti-melanogenic efficacy of Melasolv in various cellular models [18, 20] and found that Melasolv did not directly inhibit tyrosinase activity; as such, the precise regulatory mechanism underlying its mechanism of action remained unclear [18]. Melanosomes are sites of intracellular melanin synthesis, storage, and transportation that provide tissues with pigment and photoprotection [25]. In this study, we found that Melasolv activates the process of autophagy that results in increased degradation rates of intracellular melanosomes.

Among various regulatory signaling pathways that are known to contribute to the activation of autophagy, the pathway via mTOR signaling is the best defined [12, 26]. Upon activation of autophagy by starvation or treatment with an mTOR inhibitor, the mTOR complex 1 becomes inactive, resulting in dephosphorylation and nuclear translocation of TFEB; once in the nucleus, TFEB activates target genes including the ATGs [12, 26]. One of the main regulators of mTOR is adenosine monophosphate-activated protein kinase (AMPK) [26]. Consistently, our results indicate that treatment with the mTOR inhibitor, Torin1, also induces nuclear translocation of TFEB in B16F1 cells. Interestingly, we also observed that treatment with Melasolv also induces nuclear translocation of TFEB in B16F1 cells (Fig 3). These results suggest that Melasolv may activate AMPK to promote dephosphorylation of TFEB. Future studies will be performed to identify the cellular signal transduction pathways triggered by Melasolv including mTOR and TFEB contribution.

Selective autophagy provides cells with an efficient means to control the quality and quantity of cellular organelles, including mitochondria, peroxisomes, lysosomes, the endoplasmic reticulum, chloroplasts, and the nucleus [5, 27]. Recognition of target organelles by the autophagosome occurs via interactions with microtubule-associated protein 1 light chain 3 (LC3) and LC3 adaptors including the p62 protein in an ubiquitin-dependent manner [5, 27]. Interestingly, the existence of an autophagic degradation pathway that targets melanosomes had not been addressed. Here, we developed a novel system to monitor autophagic flux of melanosomes using tandem fluorescent-tagged TPC2 (TPC2-mRFP-EGFP). With this system, we found that treatment with Melasolv resulted in a dramatic increase of the red-fluorescence-positive punctate deposits within target cells (Fig 4). As such, our findings suggested that Melasolv induces melanophagy in B16F1 cells.

Generally, ubiquitination of membrane proteins of targeted organelles is required to mark them for autophagic clearance [5, 28]. For example, in mitophagy, Parkin E3 ligase enhances the recruitment of phospho-ubiquitin, promoting the ubiquitination of outer mitochondrial membrane protein such as voltage-dependent ion channel 1 and mitofusin 1/2 [5]. Levy et al. previously demonstrated that ubiquitination of a melanosome membrane protein by Homologous to the E6-AP Carboxyl Terminus (HECT)-E3 ligase is involved in lysosomal degradation of melanosome proteins [29]. Likewise, the autophagy adaptor protein p62 is a critical component of the pathways that promote degradation of intracellular organelles [30–33]. The p62 protein binds to ubiquitinated components and recruits the LC3 protein of autophagosome, resulting in autophagic degradation [34]. Therefore, further studies on p62 in melanophagy and ubiquitin-mediated degradation of melanosome proteins are needed to characterize and elucidate the underlying mechanisms.

In conclusion, here, we report that Melasolv promotes depigmentation in a melanoma cell line by activating autophagy, thereby promoting degradation of intracellular melanosomes.

## Supporting information

S1 Fig Raw blot images related to Fig 2C. (PPTX)

S2 Fig Raw blot images related to Fig 4B. (PPTX)

S3 Fig Raw blot images related to Fig 5B. (PPTX)
