# Supplementary material for: Melasolv induces melanosome autophagy to inhibit pigmentation in B16F1 cells
Source: PLoS One. 2020 Sep 17;15(9):e0239019. doi: 10.1371/journal.pone.0239019 (PMC7498095; doi:10.1371/journal.pone.0239019)
Supplement: S1 Fig — (PPTX) [file pone.0239019.s001.pptx]

## Slide 1
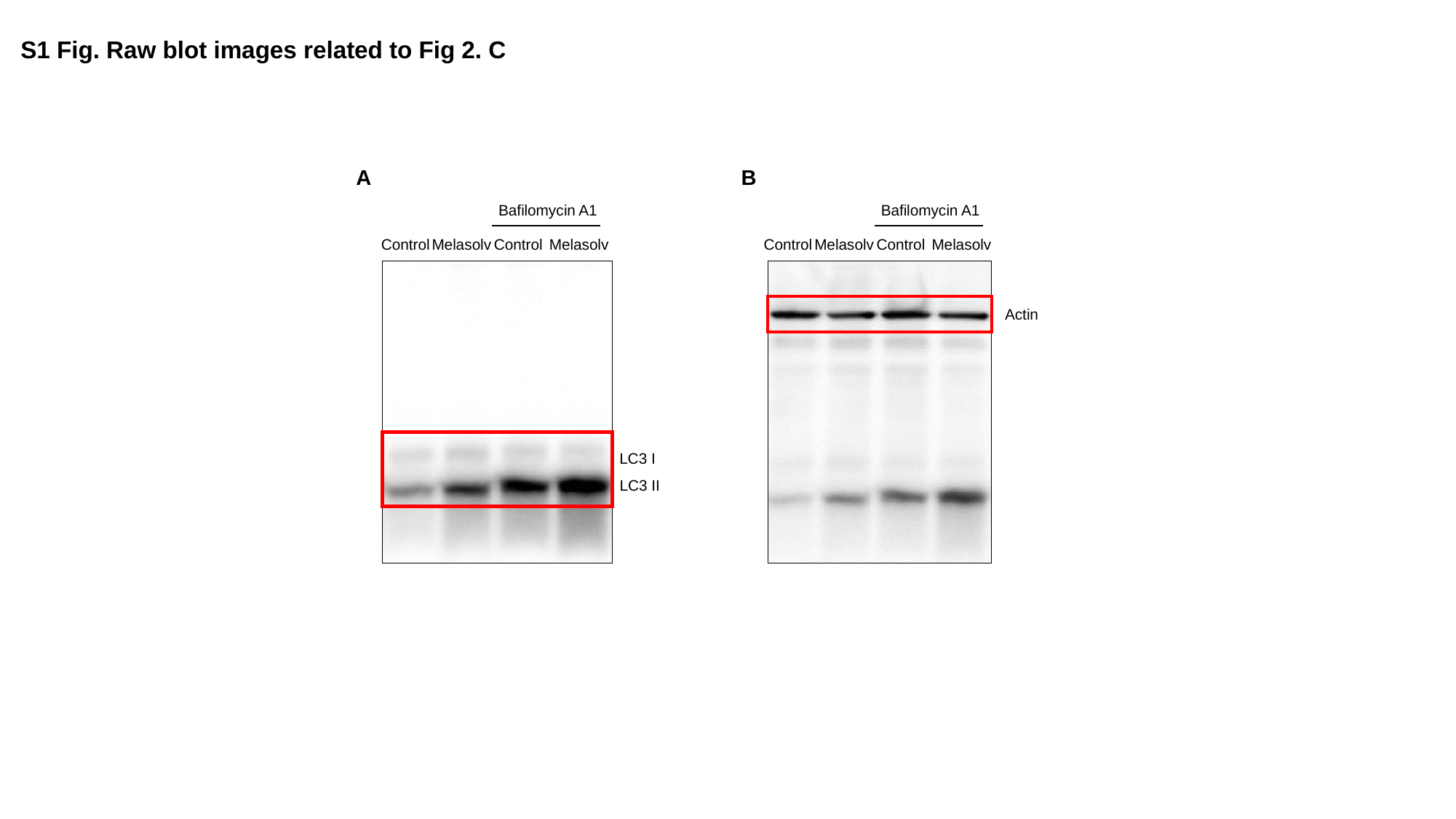

S1 Fig. Raw blot images related to Fig 2. C
A
B
Bafilomycin A1
Control
Melasolv
Control
Melasolv
Bafilomycin A1
Control
Melasolv
Control
Melasolv
Actin
LC3 I
LC3 II
