# Supplementary figures and images for: Melasolv induces melanosome autophagy to inhibit pigmentation in B16F1 cells
Source: PLoS One. 2020 Sep 17;15(9):e0239019. doi: 10.1371/journal.pone.0239019 (PMC7498095; doi:10.1371/journal.pone.0239019)

## Slide 1
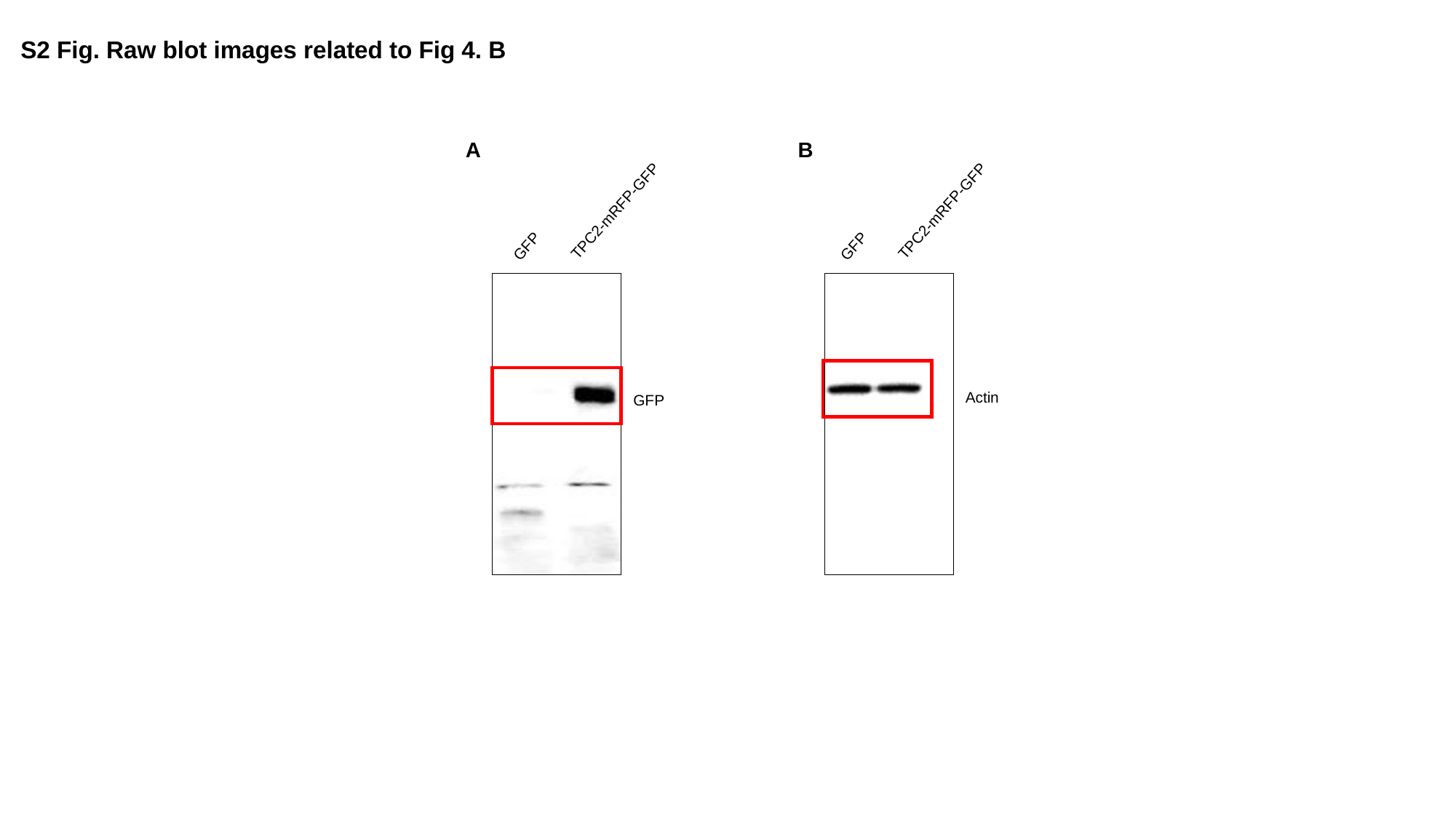

S2 Fig. Raw blot images related to Fig 4. B
A
B
TPC2-mRFP-GFP
GFP
GFP
TPC2-mRFP-GFP
GFP
Actin

Supplement: S2 Fig — (PPTX) [file pone.0239019.s002.pptx]
