# Supplementary material for: Melasolv induces melanosome autophagy to inhibit pigmentation in B16F1 cells
Source: PLoS One. 2020 Sep 17;15(9):e0239019. doi: 10.1371/journal.pone.0239019 (PMC7498095; doi:10.1371/journal.pone.0239019)
Supplement: S3 Fig — (PPTX) [file pone.0239019.s003.pptx]

## Slide 1
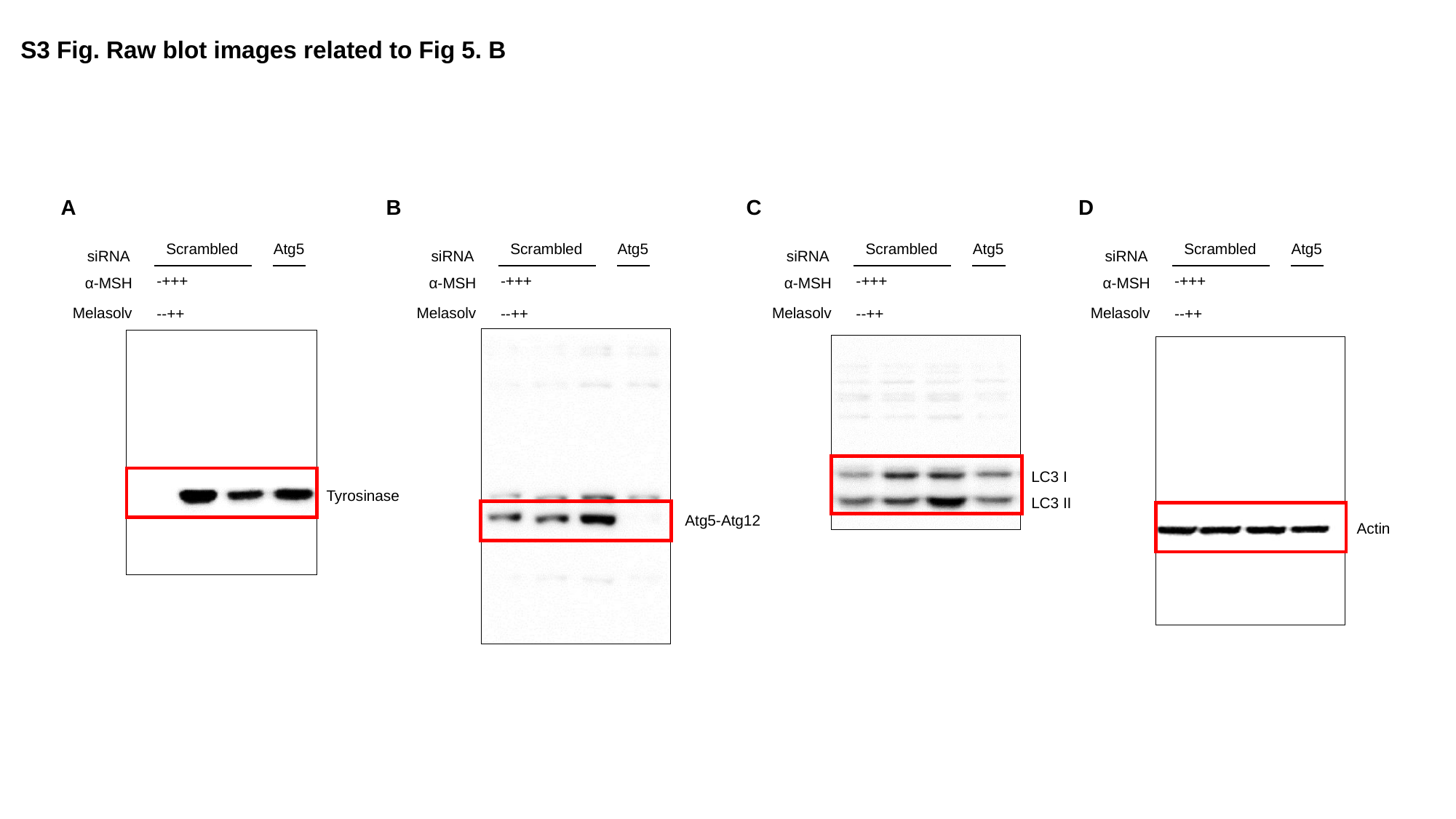

S3 Fig. Raw blot images related to Fig 5. B
A
Scrambled
Atg5
siRNA
-+++
α-MSH
Melasolv
--++
Tyrosinase
B
Scrambled
Atg5
siRNA
-+++
α-MSH
Melasolv
--++
Atg5-Atg12
C
Scrambled
Atg5
siRNA
-+++
α-MSH
Melasolv
--++
LC3 I
LC3 II
D
Scrambled
Atg5
siRNA
-+++
α-MSH
Melasolv
--++
Actin
